# Pathological changes in neurovascular units: Lessons from cases of vascular dementia

**DOI:** 10.1111/cns.13572

**Published:** 2021-01-10

**Authors:** Chao Li, Yan Wang, Xiu‐Li Yan, Zhen‐Ni Guo, Yi Yang

**Affiliations:** ^1^ Department of Neurology Stroke Center & Clinical Trial and Research Center for Stroke the First Hospital of Jilin University Changchun China; ^2^ China National Comprehensive Stroke Center Changchun China; ^3^ Jilin Provincial Key Laboratory of Cerebrovascular Disease Changchun China

**Keywords:** cerebral blood flow, neurovascular units, remote ischemic conditioning, vascular dementia

## Abstract

Vascular dementia (VD) is the second leading cause of dementia after Alzheimer's disease (AD). The decrease of cerebral blood flow (CBF) to different degrees is one of the main causes of VD. Neurovascular unit (NVU) is a vessel‐centered concept, emphasizing all the cellular components play an integrated role in maintaining the normal physiological functions of the brain. More and more evidence shows that reduced CBF causes a series of changes in NVU, such as impaired neuronal function, abnormal activation of glial cells, and changes in vascular permeability, all of which collectively play a role in the pathogenesis of VD. In this paper, we review NVU changes as CBF decreases, focusing on each cellular component of NVU. We also highlight remote ischemic preconditioning as a promising approach for VD prevention and treatment from the NVU perspective of view.

## 1 INTRODUCTION

VD is the second leading cause of dementia after AD. There is increasing evidence that vascular risk factors, such as atherosclerosis, occlusion of an artery, and small vessel disease, contribute to neurodegeneration and dementia.^1^ A previous study showed that VD accounted for a growing proportion of cases of dementia, and the disease significantly correlated with age.^2^ By the middle of the 21st century, about 40% of patients with dementia had dementia caused by chronic cerebral ischemia.^3^, ^4^ In addition to vascular problems, risk factors such as diabetes mellitus,^5^, ^6^ age, smoking, and alcohol consumption^7^ cannot be ignored.^8^ Because of its insidious onset, duration of symptoms, and lack of characteristic clinical features, it is often difficult to develop strategies for clinical prevention and treatment, despite development of imaging modalities to reduce misdiagnosis. Although AD and VD share common vascular risk factors, the mortality rate of VD is extremely high (within 3–5 years) because of cardiovascular and cerebrovascular factors.^1^ Therefore, to achieve early diagnosis of the disease and determine treatment options, an in‐depth understanding of the disease mechanism is required.

With the gradual deepening of VD studies, it becomes more and more clear that the root cause of VD is the damage of neurons and glial cells, along with alterations of axonal signal transduction and disrupted microvessels leading to energy failure,^9^, ^10^ all of which are components of NVU. Considerably, in the presence of vascular stenosis or occlusion, the integrity of the NVU is changed, resulting in VD symptoms, such as memory and spatial cognitive impairment, loss of self‐care abilities, and even death.^11^ Therefore, to explore the pathogenesis of VD from the level of NVU is a reasonable angle for VD research. In this review, we discuss the pathological changes in NVU in VD settings.

### 1.1 VD

According to the pathological changes of VD can be divided into different subtypes, the main ones are multi‐infarct dementia, small vessel dementia, strategic infarct dementia, hypoperfusion dementia, hemorrhagic dementia, hereditary vascular dementia, Alzheimer's disease with cardiovascular disease,^1^ which is mainly due to different degrees of insufficient CBF perfusion. A large sample study has confirmed that most cases of VD are caused by subcortical vascular disease rather than large cortical infarcts.^12^ At present, the diagnosis of VD mainly depends on imaging.^13^ Representative pathological changes include white matter lesion, lacunar cerebral infarction, and cerebral microhemorrhage. Although the pathological damage and cognitive impairment caused by cerebrovascular disease may be obvious, it is extremely difficult to determine the exact contribution of cerebrovascular disease to cognitive decline and dementia. Unlike AD, the predictive model of VD disease progression is still unknown, and a specific treatment is lacking.^1^ Previous studies have also shown that cerebrovascular diseases often occur along with other pathological changes, leading to cognitive impairment.^14^, ^15^, ^16^

### 1.2 Composition of NVU

NVU is composed of a neuron, glial cell, and microvessel.^17^ The vascular structure in the NVU is mainly composed of endothelial cells, pericytes, and vascular smooth muscle cells. Endothelial cells are surrounded by peripherals, astrocytes, and an extracellular matrix, forming a basement membrane. Glial cells in the NVU consist of astrocytes, microglia, and oligodendrocytes. Their main role is to provide various nutrients such as those required for nerve composition and distribution. After brain injury, glial cells are the main regulating nerve repair factors and, to a great extent, lead to the regeneration of the central and peripheral nervous systems.^18^ Pericytes located in endothelial cells, astrocytes, neurons, and the NVU are in the center of the NVU. They mainly act to integrate and process signals from neighboring cells to produce different functional responses such as regulation of the blood–brain barrier (BBB) permeability, angiogenesis, toxic metabolite removal, capillary blood flow dynamics response, nerve inflammation, and stem cell activity.^19^, ^20^

### 1.3 NVU disorders in VD

**FIGURE 1:**
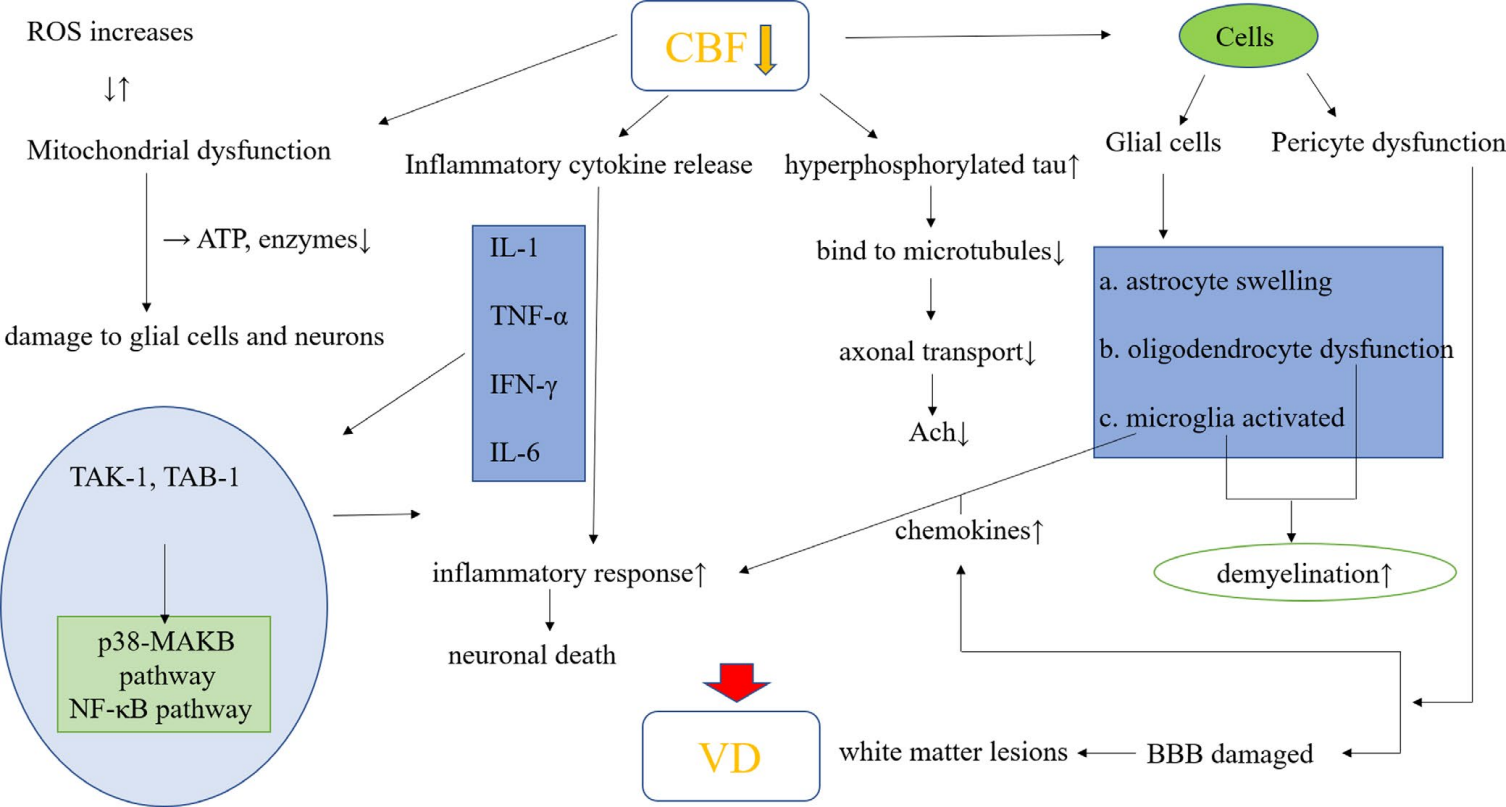
The pathological mechanism of VD induced by CBF decline

VD pathogenesis involves the following three mechanisms: (1) the decrease of CBF leads to the disturbance of energy supply to the brain, resulting in neuronal dysfunction and a series of stress reactions such as inflammation, exacerbating the neuronal damage; (2) glial cells which normally play a supporting and nutritive role in central nervous system (CNS) undergo abnormal activation and damage induced by ischemia, hypoxia and neuroinflammation; and (3) ischemia and hypoxia lead to vascular permeability changes and vascular endothelial injury.^21^ Interestingly, even in VD patients with cerebral cortical microinfarcts, global decrease in CBF is noted but perfusion around the infarction is not affected,^22^ suggesting wide‐spread NVU damage rather than local perfusion failure. This paper mainly discusses VD associated changes as a result of chronic CBF deficiency, from the NVU perspective of view (Figure 1). Nutritional supply to brain tissue by neurons and glial cells is affected, thereby resulting in VD. It is of great value to understand the pathophysiology, pathogenesis, and aggravating factors of VD, so as to develop new therapeutic targets. We also provide detailed mechanisms of how does NVU lead to the VD.

### 1.4 Mitochondrial dysfunction

Mitochondria are organelles present in eukaryotic cells, including nerve cells, and they are useful for oxidative metabolism and adenosine triphosphate (ATP) synthesis to provide energy to the cells, which is crucial for the growth and development of the cells and tissues. In addition, it is involved in important cellular processes such as apoptosis.^23^ When CBF of the CNS suddenly decreases, resulting in mitochondrial dysfunction and decrease in the energy supply and activity of ATP‐related enzymes.^24^, ^25^ The body maintains the cell energy metabolism through oxidative phosphorylation and participates in the regulation of an intracellular redox state.^26^ In addition, mitochondrial dysfunction will also lead to the production of reactive oxygen species (ROS), and an imbalance in the redox state and production of free radicals in the body will result in damage to astrocytes, VE cells, and neurons.^27^ When oxidative stress occurs, neurovascular uncoupling will occur, which further reduces CBF. Excessive ROS destroys mitochondrial function and further causes hypoxia and oxidative stress.^28^ Persistent oxidative stress may be one cause of progressive nerve injury, and excessive ROS further damages the mitochondrial function. This process repeats, thereby causing and aggravating VD. Of note, positive aspects of ROS exist such that it activates the VEGF pathway, which is involved in the formation of new blood vessels and plays a role of protecting neurons.^29^, ^30^

### 1.5 Inflammatory factors and cytokines

Increasing evidence demonstrates that post‐ischemic neuroinflammatory responses are critical in cerebral ischemia. Insufficient CBF perfusion can lead to hypoxia in brain tissue. Brain hypoxia can lead to cell death and microvascular dysfunction, and a large amount of vascular inflammatory factors may be released, thereby increasing the neuroinflammatory response.^31^ The neuroinflammatory response is mainly related to the induction of cytokines, which can be broadly classified into pro‐inflammatory and anti‐inflammatory factors. Pro‐inflammatory factors related to VD include interleukin (IL)‐1, tumor necrosis factor (TNF)‐α, interferon (IFN)‐γ, and IL‐6. IL‐1 is secreted and synthesized by astrocytes, microglia, and oligodendrocyte progenitors. Experiments have shown that IL‐1‐knockout mice cannot form myelin after brain injury, thus demonstrating that IL‐1 is mainly involved in myelin formation in the CNS.^32^ Research on IL‐1 is more thorough, and evidence shows that IL‐1 receptor and IL‐1 receptor accessory proteins form a complex form that can be activated. Interleukin‐1 receptor‐associated kinase (IRAK)‐1 and IRAK‐2 lead to downstream activation of TNF receptor‐associated factor (TRAF‐6). It will further promote transforming growth factor‐beta‐activated kinase (TAK) and TAK‐binding proteins 1 (TAB‐1) of activation, the TAB‐1 way to activate the p38‐MAKB path, eventually p38‐MAKB phosphorylation can further mediate neuron apoptosis and death.^33^, ^34^ TAK‐1 further activates the nuclear factor‐κB (NF‐κB) pathway, which is one of the most characterized transcription factors. It is widely expressed and regulates the expression of many genes, most of which encode proteins that play important roles in immunity and inflammatory processes. In the resting state, NF‐κB binds to an inhibitory protein called IκB and is present in the cytoplasm. When CBF decreases, pro‐inflammatory factors are expressed in large amounts. Among them, IL‐1, the classic activator of the NF‐κB pathway, leads to phosphorylation of IκB and further degradation by the proteasome, and NF‐κB is further transferred into the nucleus. It is important to note that activation of the NF‐κB pathway occurs in both neurons and glial cells. In the cerebral ischemia model test, TNF‐α RNA can be clearly detected after 1 hour, and TNF‐α protein increases after 2–6 hours; thus, TNF‐α responds more quickly during cerebral ischemia. Furthermore, the pro‐inflammatory role of endothelial cells and coagulant dual function aggravate ischemic head injury, leading to aggravation of cerebral microcirculation perfusion damage,^35^ which can result in severe VD. Recent studies have shown that transforming growth factor (TGF) may also be a potential harmful factor involved in VD. For example transforming growth factor‐beta 1 (TGF‐β1), produced by astrocytes and microglia mediating proliferation, differentiation, and maturation of neurons and glial cells, is significantly increased in neurodegenerative diseases.^36^, ^37^, ^38^ In a mouse VD model, increased expression of TGF‐β1 significantly reduced CBF and decreased learning ability,^39^ which further supports a detrimental role of TGF‐β1 in VD. Therefore, it is reasonable to believe that inhibiting the abnormal increase in TGF‐β1 may be an effective means to treat VD.^40^

### 1.6 Hyperphosphorylation of tau protein

Microtubules are composed of tubulin and microtubule‐related proteins. The tau protein is the most important and abundant microtubule‐related protein and plays an important role in neuronal axons, stabilizing microtubules, and inducing the correct assembly and formation of microtubules.^41^, ^42^ Dementia is significantly associated with tau hyperphosphorylation.^43^, ^44^ As CBF decreases, hypoxia may activate microglia which subsequently release free radicals and pro‐inflammatory factors. These neuroinflammatory response acts as factors driving force for neuronal p‐tau formation and even neuronal death.^45^ When tau hyperphosphorylates, it fails to bind stably to microtubules, leading to a series of neurodegenerative changes. Tau hyperphosphorylation relies on a variety of kinase activities, including cyclin‐dependent kinase 5 (Cdk5), glycogen synthase kinase 3β (GSK3), and protein phosphatase‐2A (PP2A).^46^, ^47^ Cdk5 can only bind to the neuron‐specific regulatory subunit protein p35 in cells to promote the development of neurons.^48^, ^49^ When CBF is reduced, the expression of inflammatory factors, such as IL‐1 and IL‐6, in the blood is significantly increased.^50^ First, astrocytes activate NF‐κB, which is the predominant protein significantly related to synaptic plasticity and memory. Its promoter sequences with miR‐195 negatively regulate the expression of miR‐195. The miR‐195 negative feedback regulation increases lyase‐1 amyloid precursor protein, and the increase of the amyloid protein before leading to Aβ increase in the number of synthetic beta, some Aβ across the cell membrane activated calcium protease, promote Cdk5/p35 area Cdk5/p25 expression, including Cdk5/p25 is caused by ischemia p35 truncation and formation of the fragment. Tau hyperphosphorylation is then promoted.^51^ It is worth noting that, in addition to miR‐195, miR‐126 is an indispensable part in the repair of VD. Firstly, miR‐126 can improve post‐ischemic angiogenesis, restore blood supply, and reduce neuroinflammation, thus further improving cognitive impairment. Secondly, it is also involved in maintaining the integrity of white matter and ensuring the normal operation of white matter function.^52^ Hyperphosphorylation of tau protein reduces the stable binding of tau protein to microtubules, thus disrupting axonal transport. Insufficient axonal transport results in the inability of subcellular components, such as choline acetyltransferase, in reaching the axonal end, which leads to insufficient acetylcholine synthesis at the axonal end^53^ and subsequent neurodegeneration and VD. In addition, it is well known that blood homocysteine is closely related to VD.^54^, ^55^ This has become a reliable indicator for predicting aging and pathologic cognitive dysfunction. A most recent research suggests the N‐homocysteinylation of tau and other microtubule‐associated proteins may participate in the pathological cognitive dysfunction.^56^ Moreover, this homocysteinylation process is irreversible and cannot be attenuated by folic acid and B12 supplememtation.^56^ As a result, the pathological role of tau needs more attention for future VD research.

Aside from neurons, tau protein is also expressed in oligodendrocytes in a small amount.^57^, ^58^ In the process of post‐translational modification, tau protein phosphorylation often occurs, which is crucial for the growth and development of the CNS. However, in pathological conditions like AD and VD, tau protein tends to be hyperphosphorylated, eventually leading to nerve fiber tangles and synaptic dysfunction.^59^ Ser202, Thr20, and Ser208 may be effective phosphorylation sites that promote tau protein aggregation and accelerate nerve fiber tangle formation.^60^ With decreased CBF, disturbed microglia homeostasis is associated with increased cytokine and reduced phagocytosis, further leading to larger extent of tau protein aggregation.^61^ Previous studies have shown that glial aging leads to cytokine release and promotes tau hyperphosphorylation, thus driving the development of neurodegeneration. Interestingly, senolytic agent could attenuate tau phosphorylation and cognitive function,^62^, ^63^ proving the critical role of tau hyperphosphorylation in VD pathogenesis.

### 1.7 Macroglia cell

1. Astrocytes: In addition to neurons, there are also a very important group of cells in the CNS, namely glial cells, which support and protect neurons, star BBB, and carry out substance metabolism. Astrocytes make up most of the glial cells in the CNS. Astrocytes communicate with endothelial cells to combine functions of neural activity and blood vessels in a process called neurovascular coupling. They can also respond to neuronal signals quickly in order to regulate the CBF.^64^ It has also been shown that astrocytes are involved in maintaining the integrity of the BBB and material transport.^65^ When CBF is insufficient, significant astrocyte swelling can be observed, which is the earliest response of astrocytes to ischemia and hypoxia.^66^ Glial swelling occurs mainly because ischemia leads to a lack of energy. This further leads to dysfunction or even cessation of the ion pump, which is essential for maintaining cell volume. Aquaporin (AQP)4 and AQP9 are the main AQPs.^67^, ^68^ In addition, ischemia leads to the breakdown of the BBB and development of vasogenic edema, followed by excessive uptake of protein and water by astrocytes, which ultimately leads to astrocyte swelling.^69^, ^70^ A large number of swollen glial cells can result in a brain edema, increase in intracranial pressure, and further decrease in CBF. In addition, it can also lead to the release of glutamate, which further reduces CBF.^71^, ^72^ Mild CBF is insufficient because the astrocytes in ischemic neurons are tolerant to resistance. Astrocytes can increase metabolism and confer protection from free radicals to preserve the CNS and produce protective effects. However, in dementia caused by a lack of CBF, ischemic vascular larger, longer ischemia, necrosis of neurons, and dysfunction of astrocytes will further aggravate the damage to the nervous system, thereby aggravating VD.
2. Oligodendrocytes: As one of the components of glial cells, oligodendrocytes are mainly involved in the formation of myelin sheath and maintenance of the normal function of axons of the CNS.^73^ Oligodendrocytes form lipid myelin sheaths that facilitate saltatory axonal conduction and provides metabolic and structural support to axons.^74^ Oligodendrocytes are very sensitive to hypoxia and ischemia. Reduced CBF leads to impaired oligodendrocyte function and demyelination, a pathological hallmark in VD. Apart from slowing down axonal conduction, demyelination also leads to loss of metabolic and structural support to axons, with resultant axon damage and neuronal damage.^75^ In VD, the degeneration of oligodendrocytes with aging is undoubtedly an important contributor to demyelination.^74^, ^76^, ^77^ Firstly, brain aging is associated with release of a large number of inflammatory factors that are harmful to oligodendrocytes. Secondly, oligodendrocytes are extremely sensitive to ischemia and can rapidly undergo apoptosis in the setting of chronic CBF reduction.^78^ And finally, other glial cells also participate in exacerbating oligodendrocyte injury and demyelination through releasing TIMP‐3, TNF‐α, and MMP‐3, etc.^79^ Notably, reduced myelin component production may occur independent of oligodendrocyte death.^79^

### 1.8 Microglial activation

Microglia are a kind of glial cells, which play an immune role in the CNS and act like macrophages in CNS, accounting for about 10–15% of the cells in the CNS. When the body sends out a signal of injury such as CBF decreases, microglia cells are first activated to maintain CNS homeostasis by phagocytosis of necrotic substances.^80^, ^81^, ^82^ In CNS, long‐term insufficient CBF perfusion results in white matter damage, which is a necessary structure to maintain memory and cognitive functions. White matter damage can lead to a series of degenerative changes, such as VD and AD. The central link causing white matter damage is the activation of microglia cells.^83^ Microglia cells express genes associated with cognitive impairment, such as ApoE and TREM2.^84^ In addition, they could also activate chemokines, reactive oxygen radicals, etc., which would damage axons, myelin sheath, etc., namely demyelination, a typical pathological change of VD.^85^, ^86^ Studies by Miron et al. suggest that M2‐type microglia may promote myelin regeneration to a certain extent, thereby playing a protective role against CNS.^87^

### 1.9 Changes in the BBB permeability

The BBB functions to selectively restrict substances from entering the brain; it is an important structure for maintaining a stable environment in the brain. Apart from the endothelial cells, other NVU structures such as glial end‐feet, pericytes, and subendothelial matrix, also help maintain the stability of the BBB.^88^ Destruction of the BBB is related to the dysfunction of NVU.^89^ Endothelial cell layer physically separates the brain and the blood. A balanced substance exchange between the brain and blood is crucial for the formation of new blood vessels.^90^ In addition, pericytes maintain BBB stability by secreting factors such as TGF, VEGF, and angiopoietin‐1 (ANGPT1) to facilitate endothelial cell stabilization.^91^ BBB damage has been proved to be a key pathological changes in VD, and it is worth mentioning pericyte dysfunction is an important factor aggravating VD. Evidence showed that pericyte degeneration leads to blood‐borne toxic fibrous protein leakage into the white matter, leading to a VD phenotype in mice.^92^, ^93^, ^94^ Similar finding is also observed in VD patients.^95^ The BBB is also involved in regulating the infiltration of white blood cells in the CNS. Intercellular adhesion molecule‐1 (ICAM‐1) and vascular cell adhesion molecule‐1 (VCAM‐1) play important roles in this process. These substances bind to leukocyte‐associated antigens to mediate the tight connections between astrocytes and endothelial cells.^96^, ^97^ When insufficient CBF is chronic, neuroinflammatory responses lead to the expression of a large number of cytokines and chemokines, which further promote the expression of the cellular adhesion factors ICAM‐1 and VCAM‐1.^98^, ^99^ They promote the attachment of activated neutrophils and monocytes to endothelial cells, which accumulate in large numbers in blood vessels and further reduce CBF, and can also migrate to the walls of the blood vessels.^100^ When CBF decreases causes changes in the permeability of the BBB, these inflammatory cells directly enter the brain parenchyma through the BBB and release neurotoxic substances, such as pro‐inflammatory cytokines, chemokines, and oxygen/nitrogen free radicals, thus aggravating VD.^97^, ^101^

### 1.10 Treatment prospects of remote ischemic conditioning

Remote ischemic conditioning (RIC) is a noninvasive and effectively approach whereby cuffs on the extremities are repeatedly expanded and contracted under pressures higher than the systolic pressures, based on the concept that trivial ischemia provides protection against a subsequent major ischemic event. It was first applied in patients with myocardial ischemia patients in the past 20 years.^102^, ^103^ Later, it was applied to the protect brain ischemic events. The bilateral common carotid artery stenosis model is a widely used model to reduce CBF and thus lead to VD,^104^ which can trigger an inflammatory response that ultimately leads to cell death. The mechanisms of RIC are complex and interrelated. Recent studies have shown that remote ischemic postconditioning (RIPostC) significantly increased CBF and improved cognitive impairment in bilateral common carotid artery stenosis rat model. Second, after RIPostC treatment, vascular inflammation caused by bilateral common carotid artery stenosis was significantly reduced, and the expression of ICAM‐1 and VCAM‐1 genes caused by such a pro‐inflammatory environment was increased.^105^ These substances can increase neuroinflammation and thus destroy white matter.^106^ In addition, they can downregulate the expression of glial fibrillary acidic protein in astrocytes and IBA‐1 in microglia cells, resulting in reduced white matter changes.^105^ Oligodendrocytes are mainly involved in the formation of the myelin sheath in the CNS, and the essential pathway in the development of myelin sheath is the AKT/mTOR signaling pathway.^107^, ^108^ mTOR is a key regulator of oligodendrocyte progenitor cell differentiation and myelination throughout the CNS.^109^, ^110^, ^111^ When chronic cerebral ischemia occurs, this pathway is abnormal, leading to demyelination and damage to the white matter.^112^, ^113^ Meanwhile, mTOR also can mediate atherosclerosis which can lead to CBF decrease then due to cognitive impairment. A result mTOR may be a prospective therapeutic target for VD in the future.^114^ Studies have shown that RIC induces the myelin sheath by activating the PTEN/AKT/mTOR signaling pathway, thereby protecting white matter lesions after CBF decrease.^115^

RIC also promotes anti‐inflammatory cascades and/or inhibits the synthesis of pro‐inflammatory cytokines, whereby increasing the resistance of cells or tissues to subsequent more severe ischemic events.^116^ Due to its noninvasive nature, RIC has become a practical way to treat acute and chronic neurological diseases as well as ischemic or inflammatory diseases^117^ The application of transient limb ischemia in patients with cerebral atherosclerotic stenosis can reduce the synthesis of pro‐inflammatory cytokines and increase CBF, therefore is considered as a preventive approach against stroke.^118^, ^119^ It can trigger the endogenous protective mechanism of the brain, which plays a neuroprotective role through the neural and humoral pathways. At present, some studies have confirmed the effectiveness of RIC on VD. Mi et al. demonstrated the role of distant ischemic preconditioning in increasing CBF and alleviating white matter lesions.^120^ Khan et al. demonstrated that in mice with bilateral common carotid artery occlusion, ischemic postconditioning increased CBF and behavioral outcomes.^105^ Ren et al. also demonstrated this in rats with a VD model.^121^ This may all be related to the endogenous protective mechanism of the NVU activated by RIC. Additionally, eNOS/NO has been shown to be involved in increasing CBF by promoting angiogenesis.^121^ RIC has previously been reported to protect NVU by regulating the proportion of astrocytes in the brain and weakening the activity of testosterone in astrocytes.^122^ In addition, RIC contributes to the regeneration and protection of blood vessels.^123^ This undoubtedly sheds light on RIC application in VD management strategy. In conclusion, these studies demonstrate the feasibility of RIC in light of increasing CBF for the treatment of VD.

Except that, RIC can alleviate VD through BBB pathway. CBF reduces white matter lesions were significantly associated with cognitive impairment, and patients with severe white matter changes have poor attention, executive function, and speed control.^124^ The causes of white matter changes may be related to dysfunction of the BBB, perivascular edema, and microglial cell activation.^125^ The demyelination of the white matter is further aggravated by perivascular edema caused by proteases produced by the microglial cells. At present, RIC application in patients over 80 years of age can still reduce the incidence of recurrent stroke while ensuring safety, possibly by increasing CBF and improving biomarkers of plasma coagulation and inflammation.^126^

There are no effective treatments for VD by far, and studies have shown that physical activity may improve cognitive performance.^127^ Interestingly, studies have shown that RIC acts in a similar way to exercise.^128^ Studies have shown that RIC may be more beneficial than the current medications in improving cognitive performance. Experimental studies have shown that long‐term RIC can improve cognition by enhancing spatial memory and working memory after CBF decreases.^105^, ^115^, ^129^ Therefore, this noninvasive treatment, which is pressurized through the cuff, may be useful in the future.

## 2 CONCLUSION

In this review, we analyzed the pathogenesis of VD at the NVU level focusing on each cellular component of the NVU. It is important to comprehend the VD pathogenesis from an integrated angle, since the NVU functions as a structural and function unit such that changes in one component affect the function of the entire unit. As a noninvasive modality that triggers endogenous protective mechanisms, RIC could improve VD prognosis through its influence on NVU. RIC is expected to provide a new direction for VD management.

## CONFLICTS OF INTEREST

The authors have stated explicitly that there are no conflicts of interest in connection with this article.

## Data Availability

Data available under reasonable request to the corresponding author.
